# Inappropriate Drugs in Elderly Patients with Severe Cognitive Impairment: Results from the Shelter Study

**DOI:** 10.1371/journal.pone.0046669

**Published:** 2012-10-03

**Authors:** Giuseppe Colloca, Matteo Tosato, Davide L. Vetrano, Eva Topinkova, Daniela Fialova, Jacob Gindin, Henriëtte G. van der Roest, Francesco Landi, Rosa Liperoti, Roberto Bernabei, Graziano Onder, SHELTER project

**Affiliations:** 1 Centro Medicina dell’Invecchiamento, Università Cattolica Sacro Cuore, Rome, Italy; 2 Department of Geriatrics and Gerontology, 1st Faculty of Medicine, Charles University, Prague, Czech Republic; 3 Laboratory of Research in Geriatrics and Gerontology, University of Haifa, Haifa, Israel; 4 EMGO Institute for Health and Care Research, Department of Nursing Home Medicine, VU University Medical Center, Amsterdam, The Netherlands; Cardiff University, United Kingdom

## Abstract

**Background:**

It has been estimated that Nursing Home (NH) residents with impaired cognitive status receive an average of seven to eight drugs daily. The aim of this study was to determine prevalence and factors associated with use of inappropriate drugs in elderly patients with severe cognitive impairment living in NH in Europe.

**Methods:**

Cross-sectional data from a sample of 1449 NH residents with severe cognitive impairment, participating in the Services and Health for Elderly in Long TERm care (SHELTER) study were analysed. Inappropriate drug use was defined as the use of drugs classified as rarely or never appropriate in patients with severe cognitive impairment based on the Holmes criteria published in 2008.

**Results:**

Mean age of participating residents was 84.2±8.9 years, 1087 (75.0%) were women. Inappropriate drug use was observed in 643 (44.9%) residents. Most commonly used inappropriate drugs were lipid-lowering agents (9.9%), antiplatelet agents (excluding Acetylsalicylic Acid – ASA –) (9.9%), acetylcholinesterase, inhibitors (7.2%) and antispasmodics (6.9%). Inappropriate drug use was directly associated with specific diseases including diabetes (OR 1.64; 95% CI 1.21–2.24), heart failure (OR 1.48; 95% CI 1.04–2.09), stroke (OR 1.43; 95% CI 1.06–1.93), and recent hospitalization (OR 1.69; 95% CI 1.20–2.39). An inverse relation was shown between inappropriate drug use and presence of a geriatrician in the facility (OR 0.55; 95% CI 0.39–0.77).

**Conclusion:**

Use of inappropriate drugs is common among older EU NH residents. Determinants of inappropriate drug use include comorbidities and recent hospitalization. Presence of a geriatrician in the facility staff is associated with a reduced rate of use of these medications.

## Introduction

Dementia is a common condition in institutionalized older adults: it has been estimated that in the United States (US) 50% of Nursing Home (NH) residents have a diagnosis of dementia and the majority of them are affected by other chronic diseases [1]. In this population, dementia represents a life-defining disease, in which many physical and psychological symptoms proceeded by a prolonged terminal phase might influence quality of life. In this context, a physician's care plan shifts from a curative approach to symptoms management. Notably, NH residents with impaired cognitive status receive an average of seven to eight drugs daily [2], which are often prescribed to treat chronic conditions rather than to manage symptoms, with questionable benefits to the patients [3].

Use of drugs in older adults with cognitive impairment raises several potential concerns. In particular, several studies have emphasized the need to avoid drugs that may affect cognition or induce delirium when treating patients with co-existing cognitive impairment [4]. In addition, memory loss, decline in intellectual function and impaired judgment and language, commonly observed in patients with advanced dementia, have obviously negative impact on decision making capacity, influence treatment adherence, and may cause communication difficulties including a decreased ability to report adverse effects [5], [6]. For this reason the use of drugs to treat non-dementia illnesses in older adults with severe cognitive impairment might be questionable and may lead to serious adverse effects, even when clearly beneficial drugs recommended by clinical guidelines are prescribed [5]. These concerns represent barriers to pharmacological treatment of complex patients with severe cognitive impairment and should be carefully evaluated by prescribing physicians when treating older persons with this condition [7], [8].

For this reason Holmes and colleagues have developed a set of criteria to identify inappropriate drug treatment, which can be stopped or should not be started in patients with advanced dementia [9]. The Holmes criteria were drawn by a consensus panel of experts, part of the Palliative Excellence in Alzheimer Care Efforts (PEACE) Program [10], with the purpose to decrease polypharmacy in the elderly and to reduce the use of medications that are of minimal benefit or high risk to the patients. Prevalence and factors associated with use of these drugs have been rarely evaluated in literature. The aim of the present study was to evaluate the prevalence and factors associated with the use of inappropriate drugs, as assessed by the Holmes criteria, in a sample of NH residents with severe cognitive impairment in Europe.

## Methods

### Sample and Study Setting

The Services and Health for Elderly in Long TERm care (SHELTER) study enrolled 4156 NH residents in 57 facilities of 7 European Union (EU) countries (Czech Republic, England, Finland, France, Germany, Italy, The Netherlands) and 1 non EU country (Israel). The SHELTER study has been designed to validate the interRAI instrument for Long Term Care Facilities (interRAI LTCF), a comprehensive standardized instrument, as a tool to assess the care needs and provision of care to residents in NHs in Europe [11]. The study was conducted from 2009 to 2011. In each country a sample of NHs was identified and invited to participate to the study. This sample was not randomly selected and it was not intended to be representative of all NH residents in each country. A total number of 57 NH facilities participated to the study, 10 facilities in Czech Republic, 9 in England, 4 in Finland, 4 in France, 9 in Germany, 7 in Israel, 10 in Italy and 4 in the Netherlands.

Older adults residing in participating NHs at the beginning of the study and those admitted in the 3 months enrolment period following the initiation of the study were assessed using the interRAI LTCF. In the SHELTER project no exclusion criteria were adopted. The aim of the present study was to assess use of inappropriate drugs in a sample of residents with severe cognitive impairment, defined by a CPS score of 4 to 6, admitted to NHs participating in the SHELTER project For this reason, from the initial sample of 4156 residents, those with missing data on medication use (n = 133; 3.2%), and those without severe cognitive impairment (defined as CPS<4, n = 2574; 61,9%) were excluded, leading to a final sample of 1449 NH residents. Ethical approval for the study was obtained in all countries according to local regulations. Residents were invited to take part in the study and were free to decline participation. Consent was obtained with assurance of data confidentiality.

### Data Sources

The InterRAI LTCF contains over 350 data elements including socio-demographic variables, numerous clinical items about both physical and cognitive status, as well as all clinical diagnoses, signs, symptoms, syndromes and treatments provided. The SHELTER study showed that the interRAI LTCF is a reliable instrument, which enables the creation of databases that can be used to assess and compare characteristics of NH residents across countries, languages and cultures [11].

Study researchers responsible for data collection were trained following a previously validated procedure [12]. In each country, training sessions were organised to teach study researchers how to perform the assessment using the interRAI LTCF, including the specific forms and appropriate response codes, and to develop care planning. Study researchers were trained to use a variety of information sources, such as direct observation, interviews with the person under care, family, friends, or formal service providers, and review clinical records, both medical and nursing.

### Outcome Measure

As part of the InterRAI LTCF assessment, study researchers collected information on all drugs used by the residents during the three days prior to the assessment. Researchers were instructed to derive drug data from different information sources, including physician order sheets and medication administration records. Drug information included non-proprietary and proprietary name, Anatomical Therapeutic and Chemical code of the WHO Collaborating Centre for Drug Statistics Methodology [13], formulation, dosage, frequency (number of times per day, week or month the medication is taken), and route of administration. Topical treatments and drugs taken as needed in the three days prior to the assessment were also recorded.

### Inappropriate Drug Use

To identify inappropriate drug use we adopted the criteria developed and published by Holmes et al. in 2008, which identify drugs no longer appropriate for patients with advanced dementia. These criteria facilitate discontinuation of those medications that no longer conform with the goals of care in patients with advanced cognitive deficit [9]. Definition of inappropriate drug use in patients with severe cognitive impairment (based on the Holmes et al. criteria) includes drugs rarely appropriate (rarely used in palliative care, likely to be stopped and unlikely to be started) or never appropriate (drugs with any use in palliative care that should be stopped or should not be started).

### Cognitive Status

The cognitive performance scale (CPS) was used to assess cognitive status. The CPS combines information on memory impairment, level of consciousness, and executive function, with scores ranging from 0 (intact) to 6 (severe cognitive impairment). CPS scale corresponded closely with scores generated by the Mini-Mental State Examination and neurological diagnoses of Alzheimer's disease and other dementias [14]. For the present study, in line with previous research, we assessed use of inappropriate drugs in residents with severe cognitive impairment, defined by a CPS score of 4 to 6 [15].

### Independent Variables

The demographic variables included age and gender. Functional status of residents was evaluated by the seven point MDS Activities of Daily Living (ADL) Hierarchy Scale [16]. The ADL Hierarchy Scale ranges from 0 (no impairment) to 6 (total dependence in self-care). Behavioural symptoms were deemed to be present if the resident exhibited one or more of the following symptoms in the three days prior to assessment: wandering, verbally abusive, physically abusive, socially inappropriate behaviour and active resistance of care. Falls were defined as a sudden loss of balance causing the contact of any part of the body above the feet with the floor occurring in the 90 days before the assessment. Clinical diagnoses were recorded by study researchers gathering information from the residents, the general practitioner and by a careful review of patient clinical documentation and previous medical history.

Data on participating facilities were collected with the use of a specifically designed form, which included data on facility characteristics, staff and process of care. Data on the presence of a geriatrician and a pharmacist in the facility staff as well as the presence of multidisciplinary team working in the facility were recorded.

### Statistical Analysis

Characteristics of participants were compared using ANOVA analyses for normally distributed variables, nonparametric Mann–Whitney U test for skewed variables, and chi-square analyses for dichotomous variables. Inappropriate drug use (dependent variable) was operationally defined as a dichotomous variable. Multivariate analysis of this dichotomous outcome (inappropriate drug use) was performed using logistic regression models with the generalized estimating equation approach in SAS PROC GENMOD [17]. The unit of analysis was the individual resident. The generalized estimating equation method was used to adjust for the correlation among individuals residing in the same nursing facility. This approach has been previously used for analysing clustered data of patients residing in nursing facilities [15], [18]. Age, gender, country and those variables which were associated with study outcome the univariate analysis with a p≤0.10 were simultaneously entered in the multivariate analysis. Odds ratios (OR) and corresponding 95% confidence intervals (CI) were derived from this model.

In addition, in light of recently published data suggesting a benefit related to the use of anti-dementia drugs in patients with moderate or severe Alzheimer’s disease [19], additional analyses were performed after excluding acetylcholinesterase inhibitors and memantine from drugs in the Holmes list. Analyses were performed using SAS statistical software, version 8 (SAS Institute Inc, Cary, NC).

## Results

The mean age of the 1449 older NH residents with severe cognitive impairment was 84.2 (Standard Deviation 8.9) years, 1087 (75.0%) were women. The mean number of drugs used in this sample was 6.2 (SD 3.3, median 6, IQR 4–8). Inappropriate drug use was documented in 643 (44.9%) residents. As shown in Table 1, compared with residents not using inappropriate drugs, those on inappropriate drugs had a less severe ADL impairment, a higher rate of falls, were less likely to be long stay residents (≥1 year) and had a higher number of comorbidities, including ischemic heart disease, stroke, diabetes, heart failure and pneumonia. In addition, residents on inappropriate drugs were more likely to have experienced a recent hospitalization (in the last 90 days) and less likely to live in a facility which included a geriatrician among staff relative to those receiving appropriate treatment.

**Table 1:** Sample characteristics according to inappropriate drug use.

	All n = 1449 (%)	InappropriateDrug Use* n = 643 (%)	No Inappropriate DrugUse n = 806 (%)	p
***Demographics***				
Age, years (mean±SD)	84.2±8.9	84.2±8.2	84.2±9.6	0.972
Female gender	1087 (75.0)	471 (73.3)	616 (76.4)	0.179
***Geriatric conditions***				
ADL Hierarchy Scale score (mean±SD)	4.7±1.3	4.6±1.3	4.9±1.2	<0.001
Behavioral symptoms	821 (56.1)	354 (55.1)	458 (56.9)	0.489
Falls	250 (17.4)	132 (20.7)	118 (14.8)	0.004
***Long staying (>1 year)***	978 (68.8)	417 (65.8)	561 (71.2)	0.029
***Comorbidities***				
Number of diseases (mean ± SD)	2.6±1.5	2.8±1.5	2.4±1.5	<0.001
Ischemic heart disease	355 (24.6)	183 (28.6)	172 (21.4)	0.002
Stroke	350 (24.2)	177 (27.6)	173 (21.5)	0.008
Diabetes	283 (19.6)	163 (25.4)	120 (14.9)	<0.001
Heart failure	211 (14.6)	120 (18.8)	91 (11.3)	<0.001
Cancer	133 (9.2)	65 (10.1)	68 (8.5)	0.273
Parkinson’s Disease	122 (8.4)	56 (8.7)	66 (8.2)	0.775
Urinary Tract Infections	100 (6.9)	52 (8.1)	48 (6.0)	0.118
Pneumonia	56 (3.9)	33 (5.1)	23 (2.9)	0.028
Fractures	31 (2.1)	12 (1.9)	19 (2.4)	0.586
Recent Hospitalization†	201 (13.9)	114 (17.9)	87 (10.8)	<0.001
***Facility features***				
Presence of a geriatrician	866 (59.8)	361 (56.1)	505 (62.7)	0.013
Presence of a pharmacist	454 (31.3)	209 (32.5)	245 (30,4)	0.393
Multidisciplinary Team	1333 (92.0)	592 (92.1)	741 (91.9)	0.926

ADL – Activities of Daily Living.

* includes drugs defined as never or rarely appropriate based on Holmes criteria.

† any hospitalization occurring in the last 90 days before assessment.

**Figure 1:**
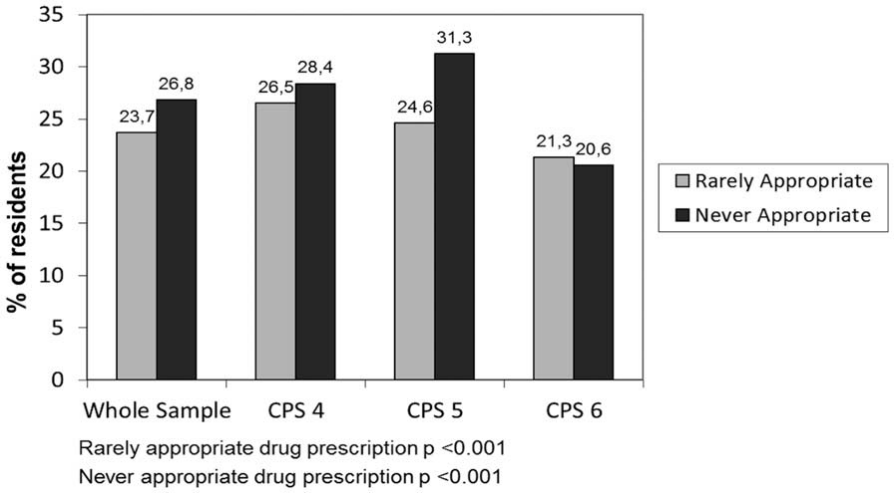
Inappropriate drug use according to Cognitive Performance Scale (CPS) score.

With regard to appropriateness of drugs prescription according to CPS score, Figure 1 shows that the prevalence of rarely appropriate and never appropriate drug prescription varied according to CPS score (p<0.001). In particular, the prevalence of use of never and rarely appropriate drugs was lower in residents with the most severe cognitive impairment (CPS = 6). Table 2 shows the pattern of inappropriate drug use according to the CPS score. Among rarely appropriate drugs antispasmodics and digoxin were the most commonly used (6.9% and 5.3% respectively), followed by anticoagulants, alpha-blockers and bisphosphonates. Among the never appropriate drugs, lipid-lowering agents (9.9%) and antiplatelet agents (excluding ASA) (9.9%) were the most commonly prescribed. Use of acetylcholinesterase inhibitors and memantine was also common in this sample (7.2% and 5.3% respectively).

**Table 2:** Inappropriate drug use according to the Cognitive Performance Scale (CPS) score.

Drug class	All n = 1449(%)	CPS 4 n = 215 (%)	CPS 5 n = 694 (%)	CPS 6 n = 540 (%)	p
**Inappropriate Drug (rarely + never appropriate)**	643 (44.4)	101 (47.0)	340 (49.0)	202 (37.4)	<0.001
**Rarely Appropriate**					
Antispasmodics	100 (6.9)	15 (7.0)	54 (7.8)	31 (5.7)	0.373
Digoxin	77 (5.3)	15 (7.0)	35 (5.0)	27 (5.0)	0.499
Warfarin	71 (4.9)	8 (3.7)	37 (5.3)	26 (4.8)	0.629
Heparin and Low-weight heparins	43 (3.3)	9 (4.2)	15 (2.2)	19 (3.5)	0.197
Alpha Bockers	41 (2.8)	9 (4.2)	21 (3.0)	11 (2)	0.250
Biphosphonates	40 (2.8)	5 (2.3)	24 (3.5)	11 (2.0)	0.292
Antiarrhythmics	33 (1.5)	0 (0.0)	13 (1.9)	9 (1.7)	0.137
Tamsulosin	20 (1.4)	5 (2.3)	11 (1.6)	4 (0.7)	0.197
Clonidine	17 (1.2)	4 (1.9)	7 (1.0)	6 (1.1)	0.590
Urinary Antispasmodics	10 (0.7)	2 (0.9)	5 (0.7)	3 (0.2)	0.847
Mineralcorticoids	4 (0.3)	2 (0.9)	0 (0.0)	2 (0.4)	0.066
**Never Appropriate**					
Lipid-lowering Medications	143 (9.9)	25 (11.6)	78 (11.2)	40 (7.4)	0.053
Antiplatelets Agents (excluding ASA)	143 (9.9)	10 (4.7)	75 (10.8)	58 (10.7)	0.021
Acetylcholinesterase inhibitors	104 (7.2)	20 (9.3)	67 (9.7)	17 (3.1)	<0.001
Memantine	77 (5.3)	16 (7.4)	48 (6.9)	13 (2.4)	0.001
Immunomodulators	4 (0.3)	3 (1.4)	1 (0.1)	0 (0.0)	0.003
Hormone Antagonists (including antiestrogens)	2 (0.1)	0 (0.0)	2 (0.3)	0 (0.0)	0.336
Cytotoxic chemotherapy	2 (0.1)	0 (0.0)	1 (0.1)	1 (0.2)	0.824
Leukotriene Receptor Antagonists	1 (0.1)	0 (0.0)	1 (0.1)	0 (0.0)	0.580

Results of the multivariate analysis are reported in Table 3. Use of inappropriate drugs (including never and rarely appropriate) was directly associated with specific diseases including diabetes (OR 1.64; 95% CI 1.21–2.24), heart failure (OR 1.48; 95% CI 1.04–2.09), stroke (OR 1.43; 95% CI 1.06–1.93), and a recent hospitalization (OR 1.69; 95% CI 1.20–2.39). An inverse relation was shown between inappropriate drug use and the presence of a geriatrician on the staff (OR 0.55; 95% CI 0.39–0.77). Only stroke was independently associated with the use of never appropriate drugs (OR 1.50; 95% CI 1.07–2.10). Finally, an inverse relation was described between likelihood of receiving inappropriate drug and the severity of functional impairment (OR 0.82; 95% CI 0.71–0.94).

**Table 3:** Factors associated with inappropriate drug use.

	Never Appropriate Drugs	Inappropriate drug use (Never + Rarely Appropriate Drugs)
	Odds Ratio (95% Confidence Interval)	
***Demographics***		
Age (10 yrs increment)	0.91 (0.79–1.05)	1.08 (0.89–1.16)
Female gender	1.15 (0.84–1.57)	0.97 (0.74–1.27)
***Long Term Stay***	0.89 (0.65–1.20)	0.79 (0.61–1.03)
***Geriatric Conditions***		
ADL hierarchy scale score	0.82 (0.71–0.94)	0.92 (0.81–1.04)
CPS score		
4	1	1
5	1.18 (0.80–1.74)	1.06 (0.75–1.51)
6	0.79 (0.50–1.26)	0.77 (0.51–1.71)
Falls	1.22 (0.87–1.70)	1.25 (0.92–1.70)
***Comorbidities***		
Number of diseases	1.04 (0.92–1.17)	1.03 (0.92–1.16)
Ischemic heart disease	1.06 (0.74–1.54)	1.20 (0.87–1.66)
Diabetes	1.39 (0.99–1.93)	1.64 (1.21–2.24)
Heart failure	0.95 (0.64–1.41)	1.48 (1.04–2.09)
Stroke	1.50 (1.07–2.10)	1.43 (1.06–1.93)
Pneumonia	1.29 (0.67–2.47)	1.47 (0.80–2.70)
Recent Hospitalization †	1.08 (0.73–1.60)	1.69 (1.20–2.39)
***Facility features***		
Presence of a geriatrician	0.73 (0.51–1.05)	0.55 (0.39–0.77)

Analyses are adjusted by Country.

ADL – Activities of Daily Living. ADL hierarchical scale score ranges from 0 (no impairment) to 6 (total dependence in self-care).

CPS – Cognitive Performance Scale score.

† Any hospitalization occurring in the last 90 days before assessment.

After exclusion of memantine and acetylcholinesterase inhibitors from the list of inappropriate drugs, use of inappropriate drugs (including never and rarely appropriate) was documented in 540 residents (37.3%). Results of the multivariate analysis showed that use of inappropriate drugs (as evaluated by these revised criteria) was associated with diabetes (OR 1.86; 95% CI 1.36–2.54), heart failure (OR 1.61; 95% CI 1.13–2.30), stroke (OR 1.72; 95% CI 1.27–2.34), recent hospitalization (OR 1.74; 95% CI 1.22–2.48) and the presence of a geriatrician on the staff (OR 0.64; 95% CI 0.45–0.91).

## Discussion

The present study shows that use of inappropriate drugs is common in NH residents with severe cognitive impairment in Europe, with about half of the study sample receiving inappropriate medications. Comorbidity, a recent hospitalization, functional impairment and presence of a geriatrician in the facility were associated with a reduced risk of inappropriate drug use. Lipid-lowering drugs and antiplatelet agents (but not ASA) followed by anti-dementia drugs (acetylcholinesterase inhibitors and memantine) were the most frequently inappropriate drugs used, followed by antispasmodics, digoxin and anticoagulants.

Use of medications is a fundamental component of the care for elderly people. The optimization of drug prescribing in this group of patients has become an important public-health issue worldwide. The aging process determines an increase of prevalence of chronic diseases and a progressive deterioration of organ function, that in turn affect the body’s ability to metabolize medicines. These alterations change drug pharmacokinetics and pharmacodynamics and increase sensitivity of older patients to adverse drugs reactions (ADRs) [20], [21]. Older adults in NHs usually suffer from multiple comorbidities, functional and cognitive impairment, geriatric syndromes and often use polypharmacotherapy. These factors may influence the efficacy of prescribed drugs and limit their benefits.

In particular, use of drugs in older adults with severe cognitive impairment represents a challenging task for prescribing physicians. As mentioned, drugs that may affect cognition or induce delirium should be avoided when treating patients with co-existing cognitive impairment [4]. In addition, patients with cognitive impairment may have communication difficulties and therefore under-report adverse effects [5], [6]. Advanced cognitive impairment may be associated with feeding problems which make the oral administration of several medications difficult [22]. Finally, cognitive impairment is associated with limited life expectancy and therefore limits the efficacy of pharmacological treatments and questions the appropriateness of multiple drug use [23], [24]. These factors pose a risk for drug related adverse outcomes of these patients and challenge the physician with difficult ethical decisions. For each patient with advanced cognitive impairment, a careful evaluation of potential benefits and risks of any prescribed medication should be performed and unnecessary or futile pharmacological treatment stopped [7], [22].

In agreement with a previous study [3], lipid-lowering agents were the inappropriate drug most commonly used in our sample. Use of these drugs might be questionable in a cohort of patients with end-stage dementia and limited life expectancy when quality of life is the main focus of care and the benefits of these drugs are irrelevant. Also use of anti-dementia drugs was common, despite their benefits in patients with severe dementia are still being debated [19], [25], [26]. Recently [26] the DOMINO trial suggested that in patients with moderate to severe Alzheimer’s dementia continuing donepezil, as compared with discontinuing it, was associated with better scores on measures of cognitive ability and activities of daily living [19]. Notably, only half of the patients who were assigned to continue donepezil in this trial maintained their treatment for the entire study period, apparently because of a perceived lack of efficacy and adverse effects [27].

Almost half of the study participants were receiving inappropriate drugs. A possible reason for this finding is the lack of a systematic recommendation for reducing unnecessary medications at the end of life, especially those used for the treatment of chronic diseases. This lack of clear guidance may lead prescribing physicians to adopt different approaches in such complex patients [7], [22]. While some physicians may believe that pharmacological treatment must follow recommendations issued for young adults to provide the best outcomes, others may be concerned regarding such an approach and believe that drug treatment should be tailored to residents’ needs, taking into consideration clinical conditions, but also non clinical factors, including function, geriatric conditions and life expectancy. Such a comprehensive approach, traditionally endorsed by geriatric medicine might result in a reduced number of prescribed drugs and explain the reduced prevalence of polypharmacy associated with presence of a geriatrician on the facility staff [28].

In addition, our findings suggest that hospitalization may lead to an increased use of inappropriate drugs among people with severe cognitive impairment, and underlie the need for a careful review of treatments after acute events causing transitions across different healthcare settings. Indeed, residents admitted to hospital often have a genuine need for many drugs and may be victim of a “prescribing cascade” which leads to an increased likelihood of receiving inappropriate drug therapy. Severe functional impairment in our sample was associated with a reduced rate of use of drugs classified as never appropriate drugs. This finding may be related to the lack of data on benefits of pharmacological treatment in disabled older adults [7], [21]. In addition, in prescribing medications the patient’s estimated life expectancy should be weighed against the time required to achieve benefit from that treatment. For this reason use of many drugs should be avoided in disabled NH residents with a limited life expectancy.

In our sample, use of inappropriate drugs seems associated with a 22% increased rate of falls, but this association does not reach statistical significance in the multivariate model. The lack of a significant association might be related to the fact that the effect of inappropriate drug use on falls is smaller than we can detect.

The present study presents several limitations. First, the severe cognitive impairment (CPS 4 to 6) as defined by the Cognitive Performance Scale does not allow us to recognize its cause. Second, only drugs taken during the three days prior to the assessment were recorded in the present study. This could lead to an underestimate of the prevalence of inappropriate drug use because several drugs may be administered weekly (i.e. bisphophonate). Third, the study sample was not meant to be nationally representative and therefore results cannot be generalized to all NH residents in Europe. Finally, to assess inappropriate prescribing we used a set of criteria developed in the US, whose application to a European population may be questionable because of potential differences in medication policy and pharmaceutical marketing.

In conclusion, this cross-sectional study showed that inappropriate drug use is common among NH residents and some factors may help to identify those with advanced dementia who are at high risk of medication burden. Number of comorbidities, recent hospitalization and level of disability (evaluated by Activities of Daily Living Hierarchy Scale) were associated with the use of inappropriate medications. Finally, the presence of a geriatrician in the facility was associated with a reduced rate of inappropriate drug use, suggesting that a geriatric approach can improve quality of prescribing. Data from this study may be useful to identify a population at risk of iatrogenic illness because of poor prescribing and to target interventions aimed at improving quality of drug use.
